# Evaluation of ultrasonic axillary dissection in preservation of intercostobrachial nerve and lymphatic sealing in breast cancer patients: Randomized controlled trial

**DOI:** 10.1016/j.amsu.2020.10.059

**Published:** 2020-11-01

**Authors:** Ahmed M.F. Salama, Ahmed M. Nawar, Mohamed E. Zayed, Mohamed S. Essa

**Affiliations:** Department of General Surgery, Benha University Hospital, Faculty of Medicine, Benha University, Benha, Egypt

**Keywords:** Harmonic, Electrocautery, Drainvolume, Axillary numbness, MRM, Modified radical mastectomy, BCS, Breast conservative surgery, ANLD, Axillary lymph node dissection, SLNB, Sentinel Lymph node biopsy, FDA, Food and drug administration, CONSORT, Consolidated Standards of Reporting Trials, UIN, Unique Identifying Number

## Abstract

**Background:**

Electrocautery has been shown to be associated with excessive serous drainage which may lead to many complications in patients with breast cancer needing dissection of the axillary lymph nodes. The Harmonic Focus could outperform electrocautery in dissection of axillary lymph nodes, resulting in shortening of the operative times and minimize postoperative complications**.** This study aims to compare the mean axillary drain production and the axillary numbness frequency in axillary lymph node dissection (ANLD) during Modified Radical Mastectomy (MRM) and breast conservative surgery (BCS) between the use of harmonics scalpel and electrocautery.

**Methods:**

This study includes 40 patients presented with early breast cancer (T1 and T2) underwent BCS or MRM in general surgery department, Faculty of Medicine, Benha University Hospital during the period from January 2017 to September 2019. The patients randomly assigned into 2 groups; group A: subjected to ANLD using Harmonic Focus tool and group B: subjected to ANLD using electrocautery. Operative time, total drainage volume, blood loss, duration of the drain and frequency of axillary numbness were recorded.

**Results:**

This study shows that using Harmonic in axillary dissection considerably reduced operating time, total drainage volume, blood loss, days of hospital stays and reduced axillary numbness level in comparison to conventional electrocautery.

**Conclusion:**

Compared to the normal electrocautery, the harmonic focus dissection has major advantages in lowering postoperative drainage, blood loss intra-operative and lower incidence of axillary numbness in breast cancer axillary dissection, without affecting operating time.

## Highlights

• Use of Harmonic Focus associated with decrease in blood loss and operative duration.
• Use of Harmonic focus also associated with decreases the total axillary drain output, the drain duration.
• Harmonic Focus reduce frequency of axillary numbness.

***Trial registration*:** Trial registered in the Thai Clinical Trials Registry (TCTR20200903004), registered on the 31 August 2020.

## 1 Introduction

Breast cancer is one of the most common malignancy and the leading cause of cancer-related death as it contributes in 14% of total deaths in females worldwide [1]. BCS is the preferred type of surgery among female patients; however, MRM still plays an essential role in cancer breast surgery. Electrocautery is the most common used tool for the dissection and hemostasis during MRM with the benefit of minimizing blood loss [2]. Several studies suggested that it could increase the incidence of postoperative complications such as wound infection, seroma and excessive drainage, resulting in postoperative delay in delivering the adjuvant therapy [3,4]. Nowadays the harmonic scalpel, commonly used in laparoscopic surgery, provides a promising prospect of dissection in MRM. This has the benefit of decreased thermal expansion, which decreases the frequency of tissue damage in comparison to that of electrocautery [5].

Harmonic energy is mechanical and it is not regulated by the impedance. Therefore, harmonic is ideally suited for patients who have an implanted cardiac pacemaker. The Harmonic Emphasis causes a breakdown of hydrogen bonds via generating an ultrasonic energy and hence formation of coagulum from the denatured protein. Sticky coagulum is formed by denatured protein. This coagulum seals lymph nodes and vessels in draining. The friction produces an internal tissue heat which welds the walls of the vessels. Simultaneous cutting and coagulation happens at a lower temperature with limited radial thermal diffusion (in contrast with electrosurgery), thus, lowers the incidence of adjacent tissue destruction than electrocautery (especially during dissection around the nerves which need to be preserved). It also causes minimum desiccation and charring together with low smoke production for visibility improvement [[5], [6], [7]]. We are trying in our study to evaluate the efficacy and safety of harmonic tool in ANLD in patients with breast cancer.

## 2 Methods

### 2.1 Study design and setting

This study is a single–centered, prospective, blind, randomized, controlled study conducted at the general surgery department in Benha University Hospital, between January 2017 and September 2019. Our Study has been reported in line with the Consolidated Standards of Reporting Trials (CONSORT) criteria [8]. Registration unique identifying number (UIN): 6137 (https://www.researchregistry.com/browse-the-registry#home/).

### 2.2 Inclusion criteria

Female patients with early (T1 or T2) cancer breast fit for both BCS and MRM.

### 2.3 Exclusion criteria

(1) Patients with carcinoma in-situ.
(2) Locally advanced breast carcinoma (T3 and T4).
(3) Metastatic carcinoma.
(4) Recurrent breast carcinoma.
(5) Patients who receive neoadjuvant chemotherapy.
(6) Breast sarcomas.
(7) Benign breast lump.

### 2.4 Methods of randomization and blinding

**Fig. 1:**
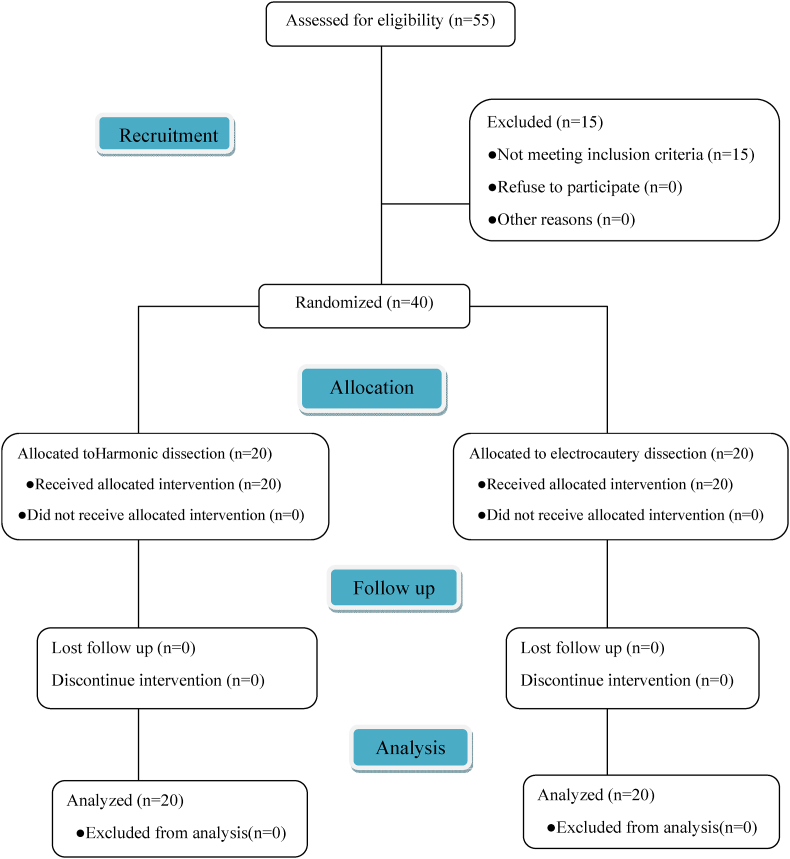
Consolidated Standards of Reporting Trials (CONSORT)flowchart demonstrating patient recruitment and exclusion.

An Excel sheet was used to create a randomization sequence with a 1:1 allocation using random block sizes of 2 and 4 by an independent doctor. A researcher who was not included with the clinical trial determined the allocation of treatment by sequentially opening numbered, opaque, sealed envelopes. The same person was also responsible after the assignment to the interventions. No patient was withdrawn from the study after randomization in addition to no changes to methods and outcomes after the commencement of the trial (Fig. 1).

### 2.5 Eligible cases

1-Group A: This group was subjected to ANLD with Harmonic Focus tool.

2-Group B: This group was subjected to ANLD with electrocautery.

### 2.6 Calculation of sample size

The sample size of the study was calculated using online software (https://clincalc.com/stats/samplesize.aspx).

After approval of the study, it was obtained by the ethical committee of the Faculty of Medicine, Benha University. This prospective randomized controlled study includes 40 female patients randomly assigned into two groups: group A, subjected to ANLD with Harmonic Focus tool, and group B, subjected to ANLD with electrocautery. After obtaining written informed consent from the patients for the participation in the study. Patients were fully informed about the hazards and benefits of the surgery. The Patients assessed by a multidisciplinary team (includes one or more specialized representative from general surgery, pathology, radiology, radiotherapy, and medical oncology) and patients enrolled in the study if they fulfilled our inclusion criteria. All patients underwent the following:

1 Full detailed history.
2 Clinical examination.
3 Laboratory investigations:

Complete blood picture (CBC), fasting and postprandial blood glucose liver function tests, renal function tests in addition to CA 15–3.

4 Radiological investigations
  (a) Bilateral mammography and ultrasonography
  (b) Metastatic work up (Computerized tomography of the chest, pelvis-abdominal ultrasound and bone scan if indicated).
5 Tissue diagnosis

All patients underwent tru-cut biopsy.

### 2.7 Operative plan

#### 2.7.1 Management of axilla

Patients who presented with clinically node negative disease (N0) were subjected to sentinel lymph node biopsy (SLNB) at the time of surgery using methylene blue. Combined retro-areolar, and peri-tumoral injection techniques were done. If the excised sentinel lymph node were negative, there was no need for ANLD and the patient excluded from the study. However, if the sentinel node was positive, ALND was done (level I and II dissection). Patients who presented initially with positive node axilla were subjected to ALND**.**

#### 2.7.2 Operative technique

**Fig. 2:**
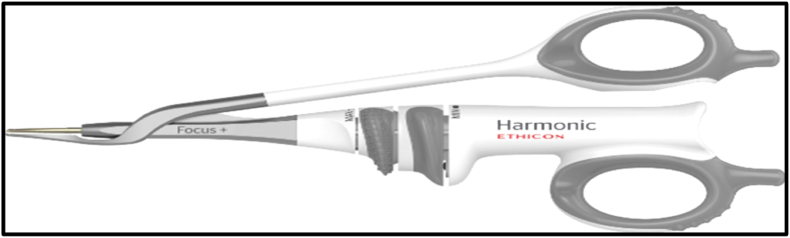
Ethicon-Endosurgeryharmonic focus.

**Fig. 3:**
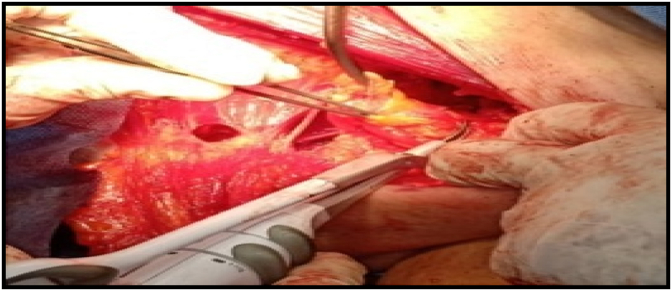
Axillary dissection using harmonic focus.

For all patients, the skin incisions were done using a scalpel; modern electrocautery was used to create the skin flaps and separate the breast together with pectoral fascia from the underlying pectoralis major muscle [Covidien (Mansfield, Massachusetts)] used with the power settings in the range of 30 W and 50 W. ANLD was done with the Harmonic Focus (Ethicon-Endosurgery HARMONIC FOCUS + Shears with Adaptive Tissue Technology)in group A using a Level 3 power system **(**Fig. 2**)**, while electrocautery was used in group B with the power settings adjusted at 30 W. Harmonic shear was used to seal lymph vessels and to provide hemostasis; no clips, sutures or electrocautery were used in Group A patients **(**Fig. 3**).**

**Fig. 4:**
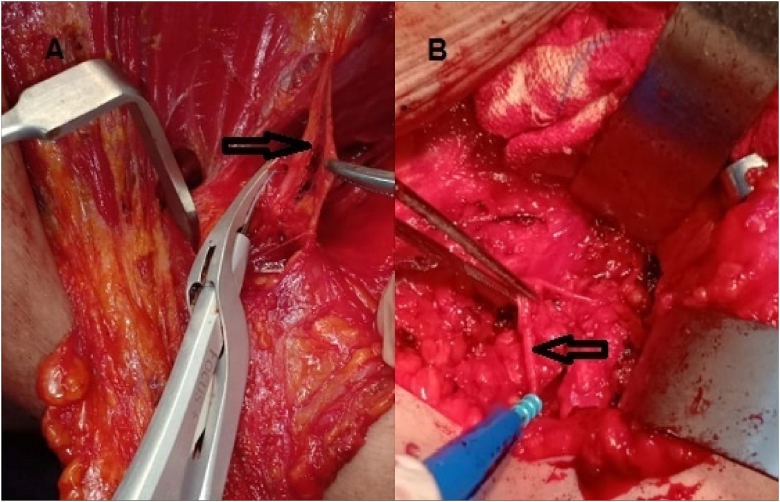
Dissection of intercostobrachial nerve (arrow):(A) dissection by harmonic focus (B) dissection by electrocautery.

Clavipectoral fascia was incisedto enter the axilla. Pectoralis major and minor were receded upwards. The axillary vein was detected, and the Harmonic sealed all of its tiny tributaries (in group A patients), while in group B patients they were ligated using 3/0 vicrylsutures. Dissection of the axillary lymph node began from the lateral side of the vein. A dissection plane was formed along the lower axillary vein border and all the fat, lymphnodes and the blood vessels from the axillary vein were dissected. The long anterior thoracic, thoracodorsal and intercostobrachial nerves were detected and protected **(**Figs. 4 **and** 5**)**.

**Fig. 5:**
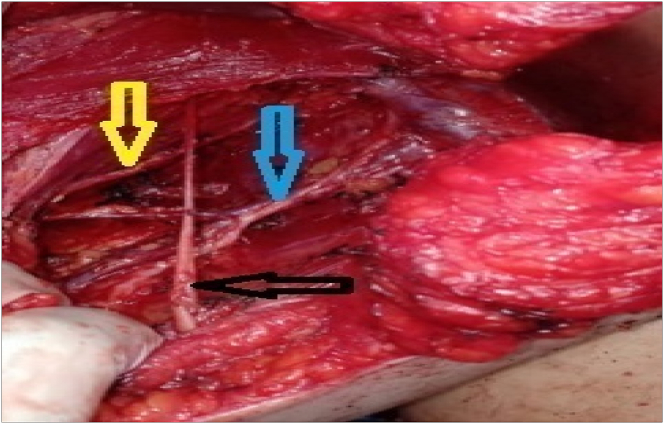
The thoracodorsal and nerve veins (blue arrow), the long thoracic.(yellow arrow) and the intercostobrachial nerve (black arrow) are retained.

Axillary dissection of level I – II was performed in all patients. Then, 1000 mL of normal saline (at 42 °C) douched the surgical area. Placement of two 16-F vacuum drains was done. All patients were treated with an antibiotic course (penicillin-based, 3-day course). Nursing workers were calculating the amount of drainage regularly. The amount of drainage was reported after discharge by the patients themselves, who endured one or more visits to offices. When the drainage volume was less than 20 mL in 24 h for two days, the drains removed.

### 2.8 Endpoints

#### 2.8.1 Primary endpoint

*Intraoperative:* operating time (from skin incision to skin closure) and blood loss (determined by sponge count and weight and suction volume).

*Postoperative:* postoperative hospital stay, total drainage volume, mean drain duration before removal, total number of lymph nodes removed and wound seroma.

#### 2.8.2 Secondary endpoint

Preservation of axillary sensation, using a piece of ice and compare the sensation with the other axilla (superficial sensation).

### 2.9 Statistical analysis

Data were coded and entered using the statistical package SPSS (Statistical Package for the Social Sciences) version 25. Data were summarized using mean, standard deviation, median, minimum, and maximum in quantitative data and using frequency (count) and relative frequency (percentage) for categorical data. Comparisons between quantitative variables were made using the non-parametric Mann-Whitney test. For comparing categorical data, Chi-square (c2) test was performed. The Exact test was used instead when the expected frequency is less than 5. P-values less than 0.05 were considered as statistically significant.

## 3 Results

This prospective study includes 40 female patients presented with early cancer breast (T1 and T2)between January 2017 to September 2019. BCS was done for 24 patients, while 16 patients underwent MRM. There was no statistically significant difference between 2 groups regarding age, BMI and tumor stage and postoperative complications **[**Table 1**].**

**Table 1:** Patients and tumors characteristics.

Variables		Group A (*n* = 20)	Group B (*n* = 20)	*P* value
Age (years)		56 ± 3	57 ± 3	0.253
BMI		26.3 ± 1.5	25.4 ± 1.9	0.192
**Tumor pathology n (%)**	**Invasive ductal carcinoma**	16 (40%)	14 (35%)	0.461
	**Invasive lobular carcinoma**	2 (5%)	1 (2.5%)	0.347
	**Paget disease of the breast**	2 (5%)	3 (7.5%)	0.319
	**Medullary carcinoma**	1 (2.5%)	1 (2.5%)	0.255
**Tumor stage**	**Stage I**	16	12	0.168
	**Stage II**	4	8	0.153
**Breast surgery**	**BCS, n (%)**	15 (37.5%)	9 (22.5%)	0.326
	**MRM, n (%)**	11 (27.5%)	5 (12.5%)	0.471
**Postoperative complications**	**Hematoma, n (%)**	0 (0%)	2 (10%)	0.562
	**Seroma, n (%)**	1 (5%)	3 (15%)	0.441
	**Wound infection, n (%)**	0 (0%)	1 (5%)	0.591
	**Flap necrosis, n (%)**	0 (0%)	0 (0%)	

### 3.1 Primary outcome (surgical outcome)

The statistical disparity between group A and group B was substantial in mean operative time (86 ± 8 min vs. 104 ± 13 min respectively; *P* ˂ 0.001). The volume of blood loss intraoperatively between the two groups was statistically significant with mean volume of 45 ± 9 mL in group A in comparison with 96 ± 27 mL in group B(P ˂ 0.001). There was statistical difference in total drain output between the two groups (847 ± 111 mL vs. 1596 ± 248 mL; P ˂ 0.001). In the harmonic focus group the number of days before the drain was removed was lower in comparison to the electrocautery group (11 ± 2 days vs. 18 ± 2 days, respectively P ˂ 0.001). There was no substantial difference between the Harmonic Focus and the electrocautery group in total nodes removed (28 ± 3 and 27 ± 3, respectively *P* = 0.341); Two Harmonic Focus patients developed seroma relative to five electrocautery patients and this was not statistically important (P = 0.407) **[**Table 2**].** There is no harms or unintended effects in both groups.

**Table 2:** Intra-operative and postoperative outcomes.

Variables	Group A (n = 20)	Group B (n = 20)	*P* value
**Operative time (min) (mean ± SD)**	86 ± 8	104 ± 13	˂ 0.001
**Blood loss (ml) (mean ± SD)**	45 ± 9	96 ± 27	˂ 0.001
**Total drain output (ml) (mean ± SD)**	847 ± 111	1596 ± 248	˂ 0.001
**Total number of lymph nodes (mean ± SD)**	28 ± 3	27 ± 3	0.341
**Mean duration of drain (days) (mean ± SD)**	11 ± 2	18 ± 2	˂ 0.001
**Wound seroma *n*(%)**	2 (10%)	5 (25%)	0.407

### 3.2 Secondary outcome (functional outcome)

Although the intercostobrachial nerve was preserved in all patients, there was four patients in harmonic focus group who developed axillary numbness compared to fourteen patients in the electrocautery group which was statistically significant with (*P* = 0.001) **[**Table 3].

**Table 3:** Frequency of axillary numbness in each group.

	Group A (*n* = 20)	Group B (*n* = 20)	*P* value
**Axillary numbness, *n* (%)**	4 (20%)	14 (70%)	0.001

## 4 Discussion

The harmonic scalpel is a revolutionary instrument that vibrates at 55.5 kHz, inducing three synergistic effects: coagulation, cavitation and cutting to achieve accurate, efficient hemostasis and dissection of tissues. This has the benefit of decreased thermal distribution, which reduces the incidence of degradation of adjacent tissue [9]. The instrument was authorized by the U.S. Food and Drug Administration (FDA) for vessel sealing with a diameter up to 5 mm [10].

This study shows that in the harmonic group, the total drain output and drain length were significantly lower than in the electrocautery group (P < 0.001). Also, the operative time and intra-operative blood loss was less in the harmonic group (P = 0.001) relative to electrocautery group. **Parveen et al** reported that axillary dissection with harmonic scalpel was safe, feasible and effective [11]. This tool simplified surgery, reduced operating time, peri-operative blood loss, drainage volume and drainage length. Additionally, the occurrences of seroma and lymphedema have decreased [12]. **Sanguinetti et al** reported the use of the harmonic scalpel in ANLD in comparison to the electrocautery, and noted a substantial decrease in blood loss and drainage length; however, no significant difference was noted in the operating time [5]**. AbulNagahet al** did a comparative study between the use of harmonic and electrocautery in MRM and concluded that the use of harmonic scalpel in MRM had shortened ANLD time and decreased the drainage volume and duration, as well as hospital stay [12].

Patient survival is not effected by preservation of the intercostobrachial nerve. It greatly avoids axillary sensory dysfunction and improves long-term symptoms [13]. In this study, 70% of electrocautery patients had axillary numbness. Although only 20% of patients were positive for axillary numbness in the harmonic scalpel side which was statistically relevant (P = 0.001). This result was in agreement with a study done by **Shuo-Hui Hung et al**, which reveal significant reduction on the number of patients with axillary numbness after their ALND in the Harmonic focus groups. This may be due to lack of risk of the electrical injury from the harmonic focus and the lower heat spread [14]. **Zu et al** reported that detection, dissection and preservation of on intercostobrachial nerve in MRM as well as in BCS were simple and straightforward. It only took 10–20 min and has the advantages of decreasing the incidence of post mastectomy pain syndrome together with improving patient quality of life following surgery significantly [15].

**Ferri E et al** reported that harmonic scalpel is effective and safe instrument in reduction of operative blood loss, operative duration, drainage volume and pain after surgery in 61 patients underwent neck dissection for head and neck cancers [16]. Meta-analysis reported by **Revelli L et al** showed that harmonic tool associated with reduction of operative duration, blood loss, postoperative pain, drainage volume and hospital stay in addition to decrease the rate of transient hypocalcaemia in comparison to conventional methods [17].

There are some limitations to this study. The procedures performed by the same surgical team, we may underestimate the amount of blood loss because the hemodilution effect during operations was not taken into consideration in addition to small number of patients enrolled in this study. In our opinion these limitations does not affect our results and conclusions regarding efficacy of harmonic tool.

## 5 Conclusion

The use of harmonic scalpel in ANLD decreases the total axillary drain output, the drain duration and reduces the frequency of axillary numbness compared to conventional electrocautery techniques. The main advantage of harmonics scalpel is its ability to achieve hemostasis and ease to use. It simplified the surgical procedure together with achieving hemostasis and efficient lymph vessels sealing and safe to use near nerves when compared with electrocautery.

## Ethical approval

All procedures performed in studies involving human participants were in accordance with the ethical standards of the institutional and/or national research committee and with the 1964 Helsinki declaration and its later amendments or comparable ethical standards.

## Consent to participate

Informed consent was obtained from all individual participants included in the study.

## Consent for publication

Written consent was obtained for publication of this study.

## Availability of data and materials

The datasets used and/or analyzed during the current study available from the corresponding author on reasonable request.

## Funding

The authors did not receive support from any organization for the submitted work.

## Authors' contributions

**Ahmed M.F. Salama:** Study conception, acquisition of data design and Drafting of manuscript.

**Ahmed M. Nawar:**Acquisition of data and literature review.

**Mohamed EZayed:**Acquisition, analysis and interpretation of data.

**Mohamed S Essa:** Drafting of manuscript and critical revision of manuscript.

## Guarantor

The corresponding author is the guarantor of submission.

## Provenance and peer review

Not commissioned, externally peer-reviewed.

## Declaration of competing interest

The authors declare that they have no conflict of interest.
